# *In Vivo* Safety of Tumor Treating Fields (TTFields) Applied to the Torso

**DOI:** 10.3389/fonc.2021.670809

**Published:** 2021-06-24

**Authors:** Roni Blatt, Shiri Davidi, Mijal Munster, Anna Shteingauz, Shay Cahal, Adel Zeidan, Tal Marciano, Zeev Bomzon, Adi Haber, Moshe Giladi, Uri Weinberg, Adrian Kinzel, Yoram Palti

**Affiliations:** ^1^ Novocure Ltd, Haifa, Israel; ^2^ Novocure GmbH, Munich, Germany

**Keywords:** Tumor Treating Fields (TTFields), cancer treatment, safety, torso, electric field simulations

## Abstract

**Background:**

Tumor Treating Fields (TTFields) therapy is a non-invasive, loco-regional, anti-mitotic treatment modality that targets rapidly dividing cancerous cells, utilizing low intensity, alternating electric fields at cancer-cell-type specific frequencies. TTFields therapy is approved for the treatment of newly diagnosed and recurrent glioblastoma (GBM) in the US, Europe, Israel, Japan, and China. The favorable safety profile of TTFields in patients with GBM is partially attributed to the low rate of mitotic events in normal, quiescent brain cells. However, specific safety evaluations are warranted at locations with known high rates of cellular proliferation, such as the torso, which is a primary site of several of the most aggressive malignant tumors.

**Methods:**

The safety of delivering TTFields to the torso of healthy rats at 150 or 200 kHz, which were previously identified as optimal frequencies for treating multiple torso cancers, was investigated. Throughout 2 weeks of TTFields application, animals underwent daily clinical examinations, and at treatment cessation blood samples and internal organs were examined. Computer simulations were performed to verify that the targeted internal organs of the torso were receiving TTFields at therapeutic intensities (≥ 1 V/cm root mean square, RMS).

**Results:**

No treatment-related mortality was observed. Furthermore, no significant differences were observed between the TTFields-treated and control animals for all examined safety parameters: activity level, food and water intake, stools, motor neurological status, respiration, weight, complete blood count, blood biochemistry, and pathological findings of internal organs. TTFields intensities of 1 to 2.5 V/cm RMS were confirmed for internal organs within the target region.

**Conclusions:**

This research demonstrates the safety of therapeutic level TTFields at frequencies of 150 and 200 kHz when applied as monotherapy to the torso of healthy rats.

## Introduction

Tumor Treating Fields (TTFields) are low intensity (1-3 V/cm RMS), intermediate frequency (100-500 kHz) alternating electric fields, displaying anti-mitotic effects on cancerous cells at cell-type specific frequencies (1, 2). TTFields (at optimal frequency of 200 kHz) have been approved by the United States Food and Drug Administration (FDA) in 2011 for the treatment of recurrent glioblastoma (GBM) and in 2015 for the treatment of newly diagnosed GBM (concomitant with temozolomide), based on the results of the EF-11 and EF-14 phase III clinical trials (3–5). TTFields also received a CE mark for treatment of GBM in Europe, and was further approved for such use in Israel, Japan and China.

Patients receive TTFields therapy continuously and non-invasively, using a portable alternating electric field (EF) generator connected to 2 pairs of arrays, which are orthogonally positioned on the patient’s skin around the tumor region to generate perpendicular fields at the tumor bed. TTFields dose, calculated based on the averages of treatment usage duration and EF intensities, was shown to positively correlate with survival outcomes in patients with newly diagnosed GBM (6–8).

To date, more than 18,000 patients with GBM have been treated with TTFields (9), and the main treatment-related adverse event reported in clinical trials and post-marketing surveillance studies has been low grade skin irritation beneath the arrays (3–5, 10, 11), which was resolved in most cases with the use of topical steroids or intermittent treatment interruptions (12). This favorable safety profile in patients with GBM is attributed to several factors: **1)** the locoregional nature of the TTFields modality, as the EF intensities outside of the brain are below the effective therapeutic intensity threshold of 1 V/cm RMS (13); **2)** the fine-tuning of the EF frequency specifically for treatment of GBM cells (200 kHz), as determined empirically (1, 2); and **3)** the low doubling rate of normal cells in the adult brain, leaving them unharmed by the anti-mitotic effect of TTFields (14).

TTFields is currently being investigated as a treatment modality for several types of solid malignant tumors that reside in the torso (15). Some of the tumors that develop in the torso are among the most aggressive cancer types, including: **1)** lung cancer, the number 1 cause of cancer-related death (approximately 1.8 million deaths globally in 2018, representing 18.4% of total cancer deaths); **2)** liver cancer, the third leading cause of cancer death (8.2%); **3)** gastric cancer, ranked third (together with liver cancer) in the number of cancer-related deaths (8.2%); and **4)** pancreatic cancer, the deadliest cancer with the lowest 5-years relative survival of 9% (16, 17). Unlike the brain, the torso contains tissues with high rates of cellular replication and turnover, such as cells of the gastrointestinal (GI) tract and the spleen, as well as circulating monocytes (14, 18). Therefore, applying TTFields to the torso could potentially interfere with replication of these normal, rapidly-dividing cells and warrants specific safety examinations.

TTFields have been applied to the torso in several phase II clinical trials at 150 or 200 kHz (according to *in vitro* determinations of optimal frequency for each type of cancer) (1, 2, 19–21). This includes studies in patients with malignant pleural mesothelioma (MPM; STELLAR trial, NCT02397928) (22), non-small cell lung carcinoma (NSCLC; EF-15 trial, NCT00749346) (23), ovarian cancer (INNOVATE trial, NCT02244502) (24), and pancreatic cancer (PANOVA trial, NCT01971281) (25). Based on the efficacy and safety observed in the STELLAR trial (22), TTFields in combination with pemetrexed and a platinum-based chemotherapy received FDA approval and a CE mark for first-line treatment of unresectable, locally advanced or metastatic MPM. In the aforementioned clinical studies, TTFields were applied in combination with chemotherapy to patients with advanced disease, which concurrently could mask any potential TTFields-specific safety issues. Accordingly, we herein describe the *in vivo* safety studies that supported the conduct of those clinical trials, in which the safety of TTFields was examined when applied as monotherapy to healthy animals at therapeutic levels (1 V/cm RMS) and optimal frequencies for torso cancers (150 and 200 kHz).

## Materials and Methods

### Animal Care

Rats were kept at room temperature (20-24°C) and 40-70% humidity, with 12 fresh air exchanges per hour. Temperature and humidity were monitored and recorded twice a day. A 12-hour light-darkness cycle was maintained throughout the experiment. Rats received standard diet with food and water ad libitum. During TTFields application rats were housed in individual cages to prevent tangling of the wires connected to the device (cage dimensions were 750 cm^2^ per rat, in compliance with international standards). For acclimation, rats were housed within the individual cages for 7 days prior to random allocation to study groups. Throughout initial arrays placement and before euthanasia, rats were anesthetized using intraperitoneal injection of ketamine (75 mg/kg) with xylazine (10 mg/kg), while light anesthesia with isoflurane was used during array replacements.

### Safety Experiment Design

The safety of TTFields application to the torso was tested in female Sprague Dawley (SD) rats (age >10 weeks, weight >200 gr, Envigo Ltd) in 2 separate sets of experiments, delivering 150 kHz TTFields or heat (sham) in the first experiment (9 animals per group) and 200 kHz TTFields or heat (sham) in the second experiment (10 animals per group). Treatments were applied continuously for a duration of 2 weeks, which is a treatment duration deemed twice as long as it takes TTFields to elicit tumor volume reductions in murine models (19–21, 26). As the clinical recommendation to maximize survival benefit for patients is to use the device continuously for at least 18 hours per day, only animals achieving that level of usage were included in the analysis (7, 8). At study end, the animals were anesthetized, blood was withdrawn, and the animals euthanized *via* intracardiac injection of 500 µl pentobarbital (200 mg/ml). After validation of animal death, post mortem procedures were performed. The study was approved by the Novocure Institutional Animal Care and Use Committee (IACUC) and the Israeli National Committee Council for Experiments on Animal Subjects. Approval numbers: 251213 and IL-18-1-3.

### TTFields Application to Animals

TTFields arrays consisted of 2 high capacitance ceramic discs (lead magnesium niobate–lead titanate [PMN-PT]) identical to those used in the clinical settings. The discs contain an internal thermistor to allow for continuous monitoring of array temperature. Sham arrays were the same physical size and shape as TTFields arrays, and included built-in resistors at the back end of the ceramic to generate heat equivalent to that produced by the treatment arrays (38.5°C). To allow and maintain optimal conductance of the arrays at the skin surface, animal fur was removed from the torso region before arrays placement, using a trimmer and depilating cream (Veet), and a layer of conductive hydrogel (identical to the hydrogel used in patients) was applied to the ceramic discs.

**Figure 1:**
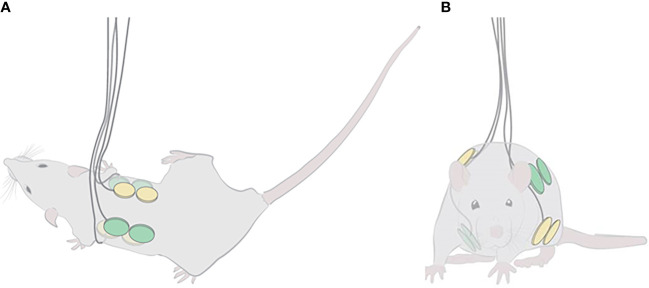
Localization of TTFields and sham arrays on the torso of rats. Illustrations of the positioning of the 2 array pairs, one shown in yellow and the other in green, on the depilated rat torso: **(A)** top view, depicting the anteroposterior positioning of the 2 ceramic discs of each dorsal array (ventral arrays may be seen transparently in the background); and **(B)** front view, demonstrating the orthogonality of the 2 array pairs.

TTFields were applied through 2 pairs of arrays, placed on the animal torso with the 2 ceramic discs of each array along the anteroposterior axis (**Figure 1A**). Arrays of the same pair were placed in opposition to one another, and the 2 pairs were positioned orthogonally, as to generate 2 roughly perpendicular electric fields for intermittent delivery (1 second in each direction) (**Figure 1B**). To obtain this configuration, the 2 dorsal arrays were placed at a distance of 1 cm from each side of the spinal cord, and the 2 ventral arrays at 5 mm from each side of the midline. Arrays were fixed to the animals using a hypo-allergenic, medical-grade adhesive covered with a flexible, plastic net to protect the wires. The wires were secured on the dorsal aspect of the rat body with upward protrusion of wires.

TTFields were applied continuously (2 weeks), utilizing the NovoTTF-100L (150 kHz) and the NovoTTF-100A (200 kHz) devices used in clinical studies with software modifications for treatment of animals. TTFields parameters were recorded continuously and stored in the log files of the device, from which device usage was determined.

### Safety Experiment Clinical Follow-Up

The rats were weighed prior to treatment initiation and at study cessation (at 2 weeks). A veterinarian conducted clinical exams twice daily throughout the treatment period for monitoring animal health. This included recording of activity level, food and water intake, stools, motor neurological status, and respiration. All adverse events were recorded.

### Safety Experiment Blood Sample Collections and Analysis

Blood samplings were performed prior to treatment initiation and at study end (at 2 weeks). For blood collection, the rats were physically restrained with the tail hanging off the edge of the counter. The tail was immersed in 42°C water for 40-50 seconds to dilate blood vessels. The tail was then wiped with 2% chlorhexidine antiseptic solution. A catheter was inserted into the vein at a shallow angle of approximately 5 cm from the tip of the tail. Then, the syringe plunger was withdrawn to collect approximately 0.8 ml of blood for further analysis.

For blood plasma, tubes that contain EDTA as an anticoagulant (10 µl of 0.1 M EDTA for 200-400 µl of blood) were used, and subsequently kept on ice. Within 10 min of collection, whole blood samples were spun at 2,000 x g in a refrigerated centrifuge (4°C) for 10 min. Plasma was collected from each sample, avoiding disruption of the red and white blood cell layers. Plasma samples were stored at 4°C until analysis of complete blood count (CBC) was performed. For blood serum, samples without anticoagulant were used and kept at room temperature for up to 30 min to enable clotting. The tubes were spun in a refrigerated centrifuge (4°C) at 2,000 x g and the supernatant collected. Serum samples were stored at -20°C, until analysis of blood biochemistry. All samples were sent on the day of collection for blinded analysis by American Medical Laboratories (AML), Israel.

### Safety Experiment Collection of Tissue Samples and Histopathological Evaluation

Postmortem macroscopic examination of muscle, fat, skin, bone marrow, heart, lung, liver, kidney, spleen, pancreas, stomach, duodenum, jejunum, ileum, cecum, colon, uterus, ovary, bladder, and brain were performed by the veterinarian. Tissue samples from each organ (except for muscle and fat, which do not contain replicating cells) were immediately dissected and placed in 4% paraformaldehyde at room temperature for 48 hr. The samples were then transferred to external independent laboratories with the appropriate expertise (PathoVet, Israel and Patho-Logica, Israel), where organs were trimmed in a standard position per organ, put in an embedding cassette and embedded in paraffin. Next, paraffin blocks were sectioned at approximately 3-5 microns thickness, the sections put on a glass slide and stained with Hematoxylin & Eosin (H&E). Pictures were taken using a microscope at magnifications of x4 and x20. The findings were semi quantitatively scored for the presence of pathological changes by the independent pathologist blinded to treatment groups according to the following scoring system: 0 = absent; 1 = minimal; 2 = mild; 3 = moderate; 4 = severe.

### TTFields Intensity Measurements

Three female Sprague Dawley (SD) rats were treated with TTFields at 150 or 200 kHz, and EF intensities in the torso were measured, using a floating scope connected to a probe inserted into the anesthetized rat abdomen. TTFields intensities were measured as a function of the current applied to the arrays (100-400 mA) in 3 different depths from the skin surface - 1, 2 and 3 cm. Linear equations describing the relation between the applied currents and the EF intensities were derived from these measurements, from which EF intensities for animals of the safety experiment were calculated, according to the specific currents recorded in the log files of the experiment (250 and 280 mA for 150 and 200 kHz, respectively, as shown in **Table 1**).

**Table 1:** Average currents (mA) applied to each pair of arrays in healthy rats treated to the torso for 2 weeks with 150 or 200 kHz TTFields.

TTFields frequency [kHz]	Animal number	Average current [mA]		Average (± SD) group current [mA]
		1st array pair	2nd array pair	
150	1	162	163	250 ± 73
	2	325	311	
	3	289	316	
	4	145	145	
	5	296	294	
	6	288	296	
	7	145	146	
	8	297	289	
	9	295	294	
200	1	281	264	280 ± 31
	2	363	269	
	3	272	254	
	4	304	311	
	5	289	229	
	6	293	287	
	7	270	279	
	8	266	294	
	9*	231	267	
	10*	318	252	

*Due to repeated TTFields treatment breaks these animals were excluded from the analysis.

### TTFields Intensity Simulations

Finite-Element Mesh (FEM) simulations were performed using the Sim4life software V5.2 (Zurich MedTech), assuming rat weight of 200 g and length of 18.5 cm (snout to vent). TTFields (150 or 200 kHz) were delivered with pairs of arrays of analogous electrical properties and spatial arrangement as the *in vivo* study. Electric properties of the various tissues were assigned according to the Sim4life software database. To simulate TTFields delivery, a constant voltage was set between the arrays to generate current intensities equivalent to those determined for the rats of the safety study (250 and 280 mA for 150 and 200 kHz, respectively). EF intensities were simulated based on the location and depths examined by the probe in the direct measurements for simulation validation. Next, intensities were simulated for the specific internal organs that were collected in the animal study for histopathological examination: heart, lung, liver, kidney, spleen, pancreas, stomach, duodenum, jejunum, ileum, cecum, colon, bladder, and brain.

### Statistical Analysis

Numerical data – blood exam results, weight, and TTFields parameters – were averaged for each experimental group, and are presented as mean ± standard deviation (SD). Each treatment group (150 and 200 kHz) was compared to its corresponding sham control group, using a Student t-test with an alpha level of <0.05 considered a significant difference. Statistical significance was calculated using GraphPad Prism 8 software (La Jolla). Results from clinical examinations and post-mortem analyses are presented descriptively.

## Results

### Device Usage

The clinical recommendation for patients treated with TTFields is to use the device continuously for at least 18 hours a day (7). Therefore, only rats of the safety experiments that had reached this threshold level (≥75% of time) were included in the analyses. Usage was determined from the active monitoring stored in the log files of the device. Daily usage of ≥18 hours per day was reached for all 9 animals in the 150 kHz group. In the 200 kHz treatment group, required usage was reached for 8 out of 10 animals, as 2 rats experienced repeated treatment breaks due to technical reasons and were thus excluded from the analysis.

### Animal Survival

All rats from the 150 kHz TTFields treatment group and corresponding sham control arm (9 animals in each) survived the study. In the second experiment, 1 animal death was reported in the control group as a result of anesthesia during sham heat array placement (prior to treatment initiation). The other 9 rats from the control arm and all 10 rats from the TTFields group (the 8 that reached required device usage as well as the 2 that were excluded from the analyses) survived the study. Overall, there was no treatment-related mortality for either the 150 or 200 kHz TTFields treated groups.

### Weight Change and Physical Status

**Figure 2:**
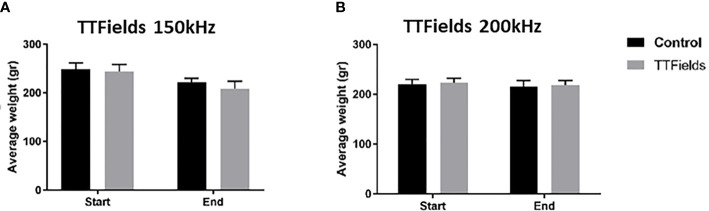
Animal weight at the beginning and end of 2 weeks of sham or TTFields application. Average weights ± standard deviations at study start and end are shown for control animals versus animals treated with TTFields at frequencies of 150 kHz **(A)** or 200 kHz **(B)**. TTFields *vs* control non-significant; Student’s t-test.

In the first experiment, in which 150 kHz TTFields were applied, average animal body weight was reduced from treatment start to cessation by 10% and 14% for rats from the control and TTFields arms, respectively (**Figure 2A**). This reduction was attributed to the general stress response associated with treatment application (i.e., attachment of arrays to animal torso that restricted motility throughout study). For the second experiment, when applying 200 kHz TTFields, the adhesive used to secure the arrays and device cables were improved and enabled enhanced animal motility. Indeed, the average reduction in body weight observed in this experiment for rats in control and TTFields groups was only 2% (**Figure 2B**). Collectively, there were no significant differences in weight loss for treatment versus control animals. In addition, no changes were observed between control rats and rats treated with either 150 or 200 kHz TTFields for activity level, food and water intake, stool, motor neurological status, or respiration.

### Complete Blood Count (CBC) and Blood Biochemistry

**Figure 3:**
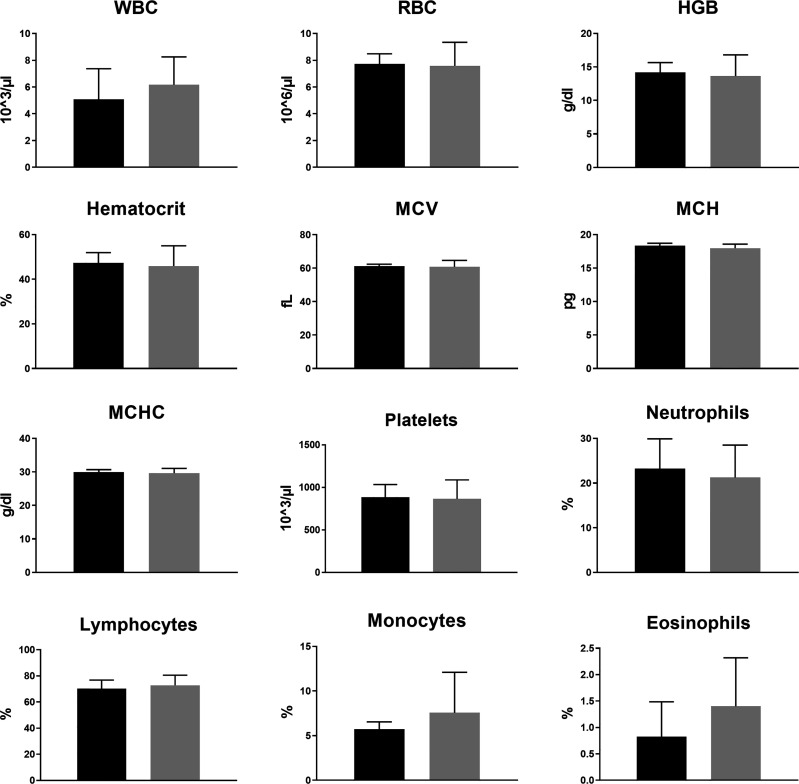
Animal complete blood count (CBC) at the end of 2 weeks of sham or 200 kHz TTFields application. Average ± standard deviations at study end are shown for control animals versus animals treated with TTFields. TTFields *vs* control non-significant; Student’s t-test. (WBC, white blood cells; RBC, red blood cells; HGB, hemoglobin; MCV, mean corpuscular volume; MCH, mean corpuscular hemoglobin; MCHC, MCH concentration).

**Figure 4:**
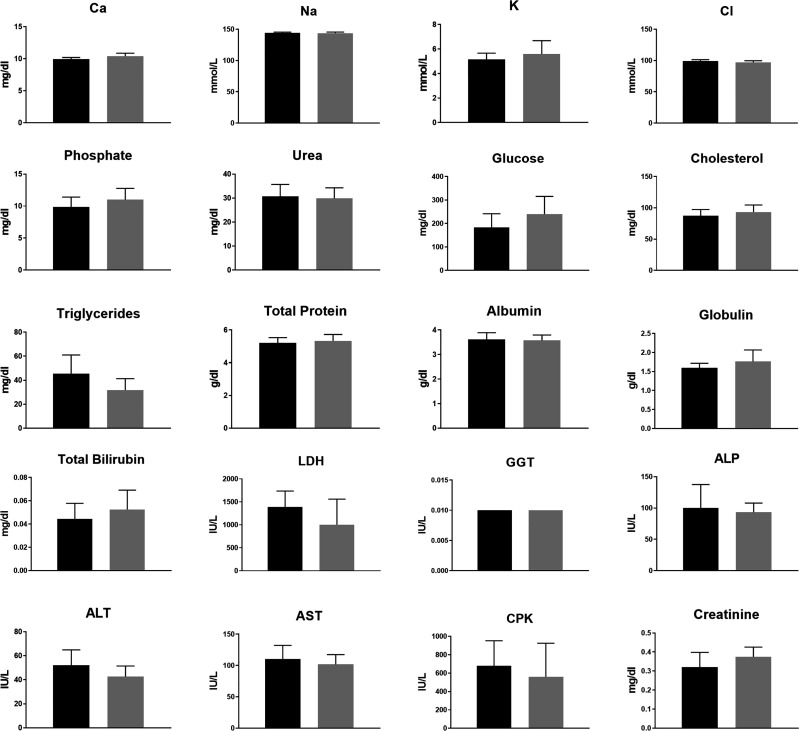
Animal blood biochemistry at the end of 2 weeks of sham or 200 kHz TTFields treatment. Average ± standard deviations at study end are shown for control animals *versus* animals treated with TTFields. TTFields vs control non-significant; Student’s t-test. (LDH, lactate dehydrogenase; GGT, gamma-glutamyl transferase; ALP, alkaline phosphatase; ALT, alanine aminotransferase; AST, aspartate aminotransferase; CPK, creatinine phosphokinase).

No major differences were observed in CBC (**Figure 3** and **Supplemetary Figure S1**) or in blood biochemistry (**Figure 4** and **Supplemetary Figure S2**) between the control and the groups treated with TTFields, except for GGT levels in the 150 kHz group. GGT levels were variable throughout that study with high levels seen before treatment initiation. As no other blood tests were abnormal, and no evidence of GGT elevation was seen in the 200 kHz study, we conclude that the observed effect was not due to a safety concern associated with treatment. Importantly, no cases of lymphopenia were reported and there were no significant changes in creatinine or electrolyte levels over time. A small increase from study inception was observed in urea levels in control and treated groups. This effect was associated with slight dehydration as a result of the constant heat generated during treatment.

### Histological Evaluation

**Figure 5:**
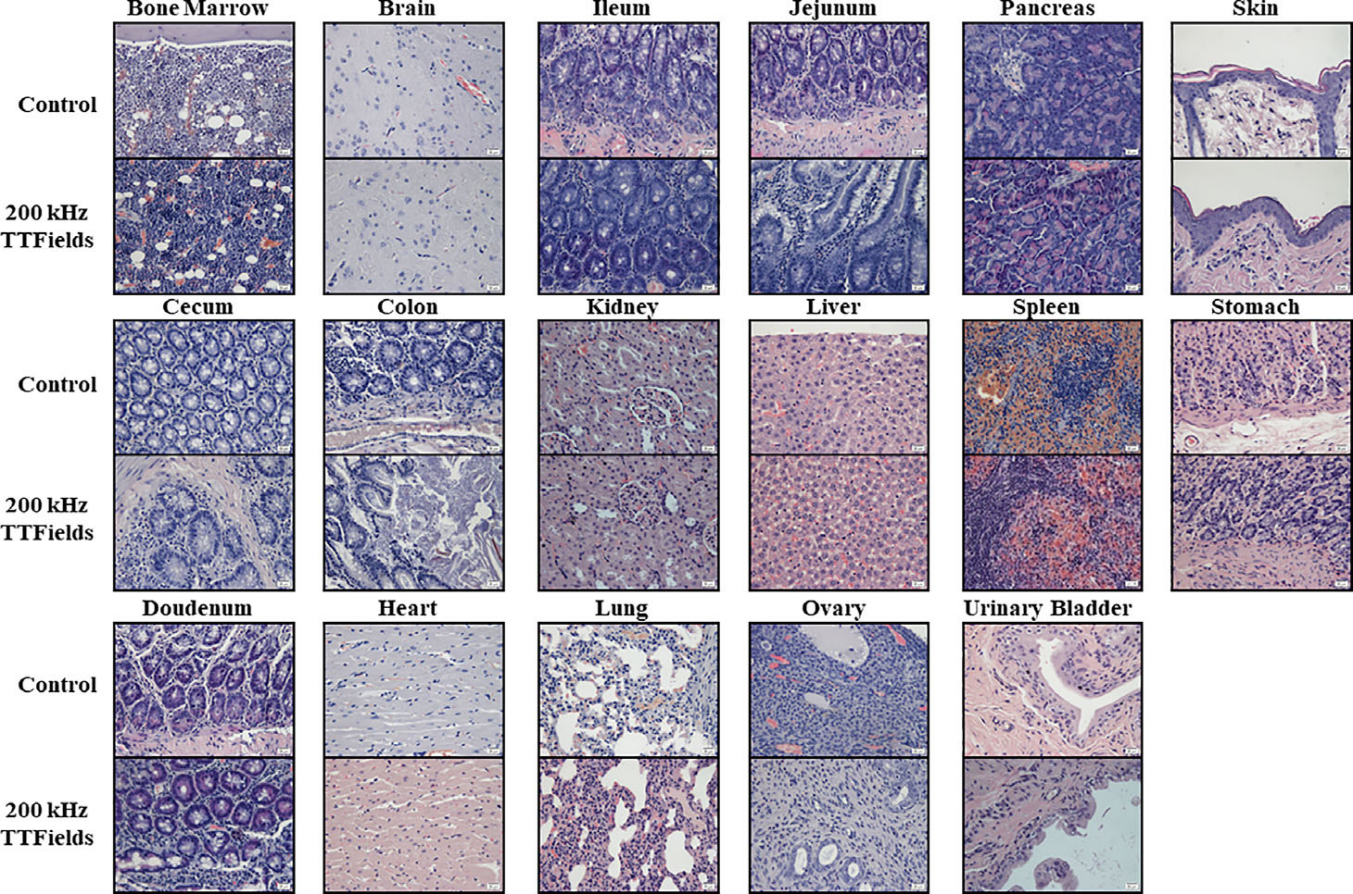
Histopathology analysis of internal organs from animals at the end of 2 weeks of sham or 200 kHz TTFields treatment. Images from one representative animal from each group are shown (H&E staining, ×20 magnification).

Histopathologic analyses of tissue samples did not reveal any morphological cytotoxic changes, signs of inflammation or other pathological changes in any tested organs from animals in control and 150 or 200 kHz TTFields groups, with a score of zero assigned by the pathologist to all examined organs from all animals (representative images are shown in **Figure 5** and **Supplemetary Figure S3**). Very mild increase of lymphocytes within the sinusoids of the liver was noted in 5 animals from the control group and 1 from the 200 kHz TTFields group. However, no cytotoxic changes to hepatocytes or changes in blood markers associated with liver dysfunction such as increased total protein, total bilirubin or liver enzymes (ALT, AST, and ALP), or reduced albumin, were observed, indicating no harm was imposed on the liver.

### Simulated Electric Fields Intensities Delivered to Internal Organs of TTFields-Treated Rats

As it is not feasible to conduct invasive EF intensity measurements in clinical trials (nor in all animal target organs), computer simulations are a valuable tool to calculate the EF intensities generated within specific organs. The first step towards simulating the intensity of TTFields delivered to internal organs was to validate the methodology, by comparing actual measured values with simulated values at each location evaluated. EF intensities were measured at various depths (1, 2, and 3 cm) and currents (100-400 mA) in the abdomen of rats treated with 150 or 200 kHz TTFields. Based on these measurements, equations were derived to correlate EF intensities (E, V/cm RMS) with applied currents (I, mA):

**Table:** 

	150 kHz:	200 kHz:	
	E(1 cm) = 0.00544·I,	E(1 cm) = 0.00568·I,	
	E(2 cm) = 0.00480·I,	E(2 cm) = 0.00455·I,	
	E(3 cm) = 0.00466·I	E(3 cm) = 0.00349·I	

**Figure 6:**
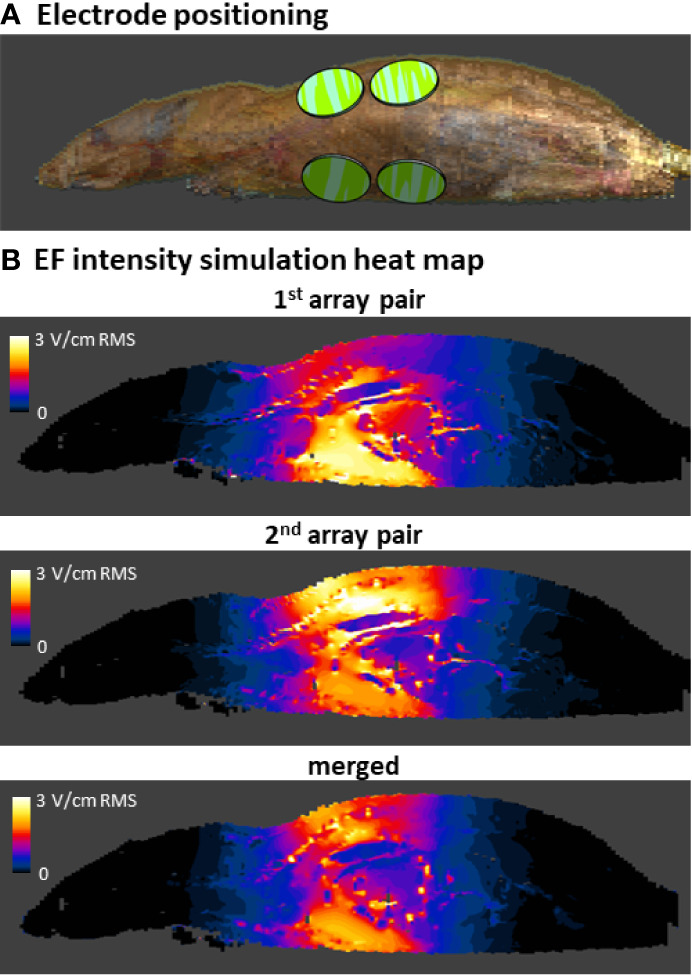
Simulations of EF intensities in the rat torso during 200 kHz TTFields application. Arrays were positioned on the rat model in accordance with their position in the safety study (only 1 side of the animal with 2 arrays is visible) **(A)**. EF intensity distribution was simulated for 200 kHz TTFields at the current determined relevant from the *in vivo* study of 280 mA, and is shown from a side view for each of the 2 array pairs and as a merged average image of both **(B)**.

The average currents measured in rats treated with 150 or 200 kHz TTFields during the 2 weeks of safety study were 250 ± 73 and 280 ± 31 mA, respectively (**Table 1**). These values were used to calculate EF intensities at the various depths based on the aforementioned empirical equations and demonstrated good agreement with the simulated values (**Table 2**), supporting further use of the simulations method to calculate EF intensities within specific internal organs (**Table 3**). Simulated EF intensities were found to range between 1.0 to 2.5 V/cm RMS for both TTFields frequencies examined in the organs situated between the arrays, which included the heart, lung, liver, kidney, spleen, pancreas, stomach, duodenum, jejunum, ileum, and cecum. For the distal organs (brain, colon, rectum, and bladder), intensities were very low (≤ 0.1 V/cm RMS). Representative distribution maps of the simulated EF intensities in the rat torso are shown in **Figure 6** and **Supplemetary Figure S4**, clearly demonstrating the locoregional nature of the treatment, with relevant EF intensities generated only between the arrays.

**Table 2:** Average EF intensities (V/cm RMS) at 1, 2, and 3 cm depth into the abdomen of healthy rats treated to the torso with 150 kHz (250 mA) or 200 kHz (280 mA) TTFields, based on direct measurements or computer simulations.

Depth [cm]	150 kHz TTFields		200 kHz TTFields	
	Measured (± SD) EF intensity [V/cm RMS]	Simulated (± SD) EF intensity [V/cm RMS]	Measured (± SD) EF intensity [V/cm RMS]	Simulated (± SD) EF intensity [V/cm RMS]
1	1.36 ± 0.07	1.41 ± 0.05	1.59 ± 0.09	1.62 ± 0.17
2	1.20 ± 0.20	1.25 ± 0.05	1.28 ± 0.21	1.31 ± 0.05
3	1.16 ± 0.19	1.08 ± 0.03	0.98 ± 0.13	1.04 ± 0.06

Currents were selected for each frequency based on the results of **Table 1**.

**Table 3:** Simulated average EF intensities (V/cm RMS) of internal organs of healthy rats treated to the torso with 150 kHz (250 mA) or 200 kHz (280 mA) TTFields.

Organ	Simulated EF intensity [V/cm RMS]	
	150 kHz TTFields	200 kHz TTFields
Brain	0.02	0.01
Heart	1.41	1.50
Lung	1.54	1.62
Kidney	1.66	1.79
Liver	2.38	2.46
Spleen	2.07	2.25
Stomach	1.38	1.49
Pancreas	1.17	1.25
Cecum	1.38	1.53
Duodenum	1.25	1.05
Jejunum	1.05	0.98
Ileum	1.22	1.03
Bladder	0.11	0.11
Colon	0.08	0.07
Rectum	0.01	0.01

Currents were selected for each frequency based on the results of **Table 1**.

## Discussion

The current study describes examinations of the safety of delivering 150 or 200 kHz TTFields as monotherapy for 2 weeks to the torsos of healthy rats. It was important to examine the conditions used in this study, to verify the delivery of therapeutic intensity TTFields. Since measuring EF intensities in all internal organs is not feasible, simulations are used to fill that gap. The simulation outcomes were first compared with actual EF probe measurements in the torsos of TTFields-treated healthy rats. These were found to be concordant with each other, validating the use of the simulations method for assessing EF intensities in the various internal organs within the torso. Such simulations predicted average EF intensities to be above therapeutic threshold of 1 V/cm RMS in organs located in the treatment target area: heart, lung, liver, kidney, spleen, pancreas, stomach, duodenum, jejunum, ileum, and cecum. In accordance with the locoregional nature of TTFields, the intensities measured at organs distant from the arrays – brain, colon, rectum, and bladder – were negligible. Altogether, these results indicate that the animal studies were indeed performed at clinically relevant TTFields intensities, and that the major internal organs of the torso of the examined healthy animals were exposed to TTFields at therapeutic doses, making the study suitable for capturing any potential safety concerns.

No treatment-related mortality, nor treatment-induced changes in activity level, food and water intake, stools, motor neurological status, respiration, or weight were observed in the *in vivo* study. In addition, no differences indicating safety concerns were observed in CBC or blood biochemistry between the active- and sham-treatment groups, nor for each group between study start and end. Furthermore, histological analyses of internal organs did not reveal any pathological findings in the TTFields-treated groups. Overall, TTFields treatment at therapeutic conditions (intensities and frequencies) showed no apparent negative effects on cells and tissues of the torso. It should be mentioned, however, that the 2 weeks duration of the study allowed capturing only acute and not cumulative long-term effects of the treatment. Furthermore, this study was performed in naïve animals, while the presence of a tumor may induce tissue remodeling and inflammation, which could potentially impact the safety and tolerability of TTFields. Nevertheless, clinical studies conducted to date with TTFields in patients with torso malignancies have confirmed the safety profile of TTFields and did not reveal any new safety signal beyond the *in vivo* findings reported here except for low grade skin irritation (27).

Cancer cells are characterized by abnormal proliferation, which rationalizes cytotoxic cancer therapies targeting cells with high basal rates of replication (28). However, these therapies also affect normal cells with higher rates of proliferation, such as cells from hair follicles, skin, and the GI tract mucosa (28). Normal GI tract organs containing cells with high rates of turnover include: stomach (2.8 days for both humans and rats), duodenum (1.5 and 1.9 days for humans and rats, respectively), jejunum (2.2 days for rats), and ileum (1.4 days for rats) (18). It is reasonable to assume that within the 2 weeks duration of this safety study, cells of the GI tract divided 5-10 times, while exposed to therapeutic intensity TTFields. Nevertheless, histopathological analysis did not reveal any changes in the healthy tissue such as aberrant mitotic figures, abnormal chromosome segregation, cellular multinucleation, and cell death previously associated with TTFields manifestation in cancer cells (29). Cells of the spleen also display relatively high turnover rates (7.8 days for humans) (14), and did not display any cytotoxic changes.

An in-depth look into the anti-mitotic mechanism of action behind TTFields is essential for understanding this positive outcome. TTFields apply directional forces on polarizable intracellular elements, and induce dielectrophoretic forces within the cell due to non-uniform fields in the cleaving mitotic cell (1). Both effects are dependent upon the frequency of the applied EF (30, 31), as supported by multiple reports demonstrating TTFields’ anti-proliferative effects to be frequency-specific for different cancer cell types (2, 20, 29, 32, 33), with the optimal frequency dependent upon cell morphology and inversely related to cell size (2, 34). Morphology deviations associated with cancer cells with respect to normal cells include changes in nuclear shape and chromatin, and cellular variability in nucleoli shape, number, and/or size (35, 36). Cancerous cells have also been suggested to differ from normal cells in the electrical properties of the cell and nucleus membranes (37, 38). These morphological and electrical differences suggest possible explanations for TTFields specificity, making cancerous cells susceptible to alternating electric fields of 150 and 200 kHz, whereas normal cells remain unharmed.

In conclusion, the results of this *in vivo* study demonstrate that TTFields at frequencies of 150 and 200 kHz can be safely applied to the torsos of healthy rats without adverse effects, despite the presence therein of tissues with high rates of cellular proliferation. This phenomenon may be attributable to the frequency specificity of TTFields for tumor cells, owing to the different morphological and electrical properties of cancerous relative to non-cancerous cells. The data from this preclinical study, together with aforementioned phase II clinical trials, led to ongoing phase III clinical trials: PANOVA-3 for locally advanced pancreatic cancer (NCT03377491), INNOVATE-3 for ovarian cancer (NCT03940196), and LUNAR for lung cancer (NCT02973789) (15). Phase II clinical studies for examining the efficacy of TTFields for treatment of gastric cancer (EF-31, NCT04281576) and hepatocellular carcinoma (HEPANOVA, NCT03606590) are also in progress (15).

## Data Availability Statement

The original contributions presented in the study are included in the article/**Supplementary Material**. Further inquiries can be directed to the corresponding author.

## Ethics Statement

The animal study was reviewed and approved by Novocure Institutional Animal Care and Use Committee (IACUC) and The Israeli National Committee Council for Experiments on Animal Subjects.

## Author Contributions

All authors contributed to study conception and design, and to data analysis and interpretation. RB, SD, MM, AS, and SC performed the animal experiments and collected the data. AZ, TM, and ZB performed the simulations. AS, SC, AH, MG, UW, AK, and YP contributed to writing, reviewing and editing of the manuscript. All authors contributed to the article and approved the submitted version.

## Conflict of Interest

Authors RB, SD, MM, AS, SC, AZ, TM, ZB, AH, MG, UW, and YP were employed by company Novocure Ltd. Author AK was employed by company Novocure GmbH.
