# Supplementary material for: In Vivo Safety of Tumor Treating Fields (TTFields) Applied to the Torso
Source: Front Oncol. 2021 Jun 24;11:670809. doi: 10.3389/fonc.2021.670809 (PMC8264759; doi:10.3389/fonc.2021.670809)
Supplement: Supplementary file 1 [file Presentation_1.pptx]

## Slide 1
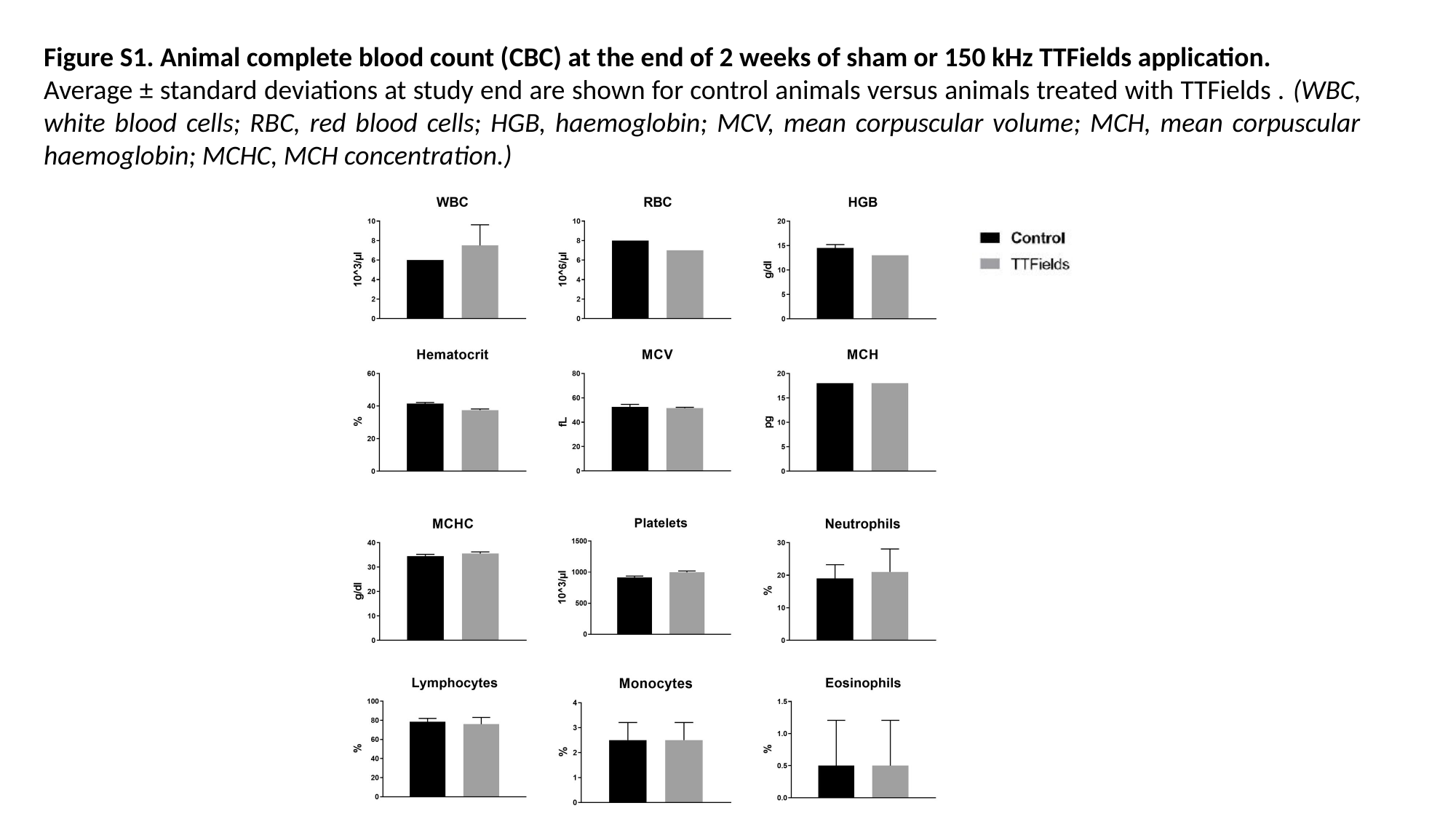

Figure S1. Animal complete blood count (CBC) at the end of 2 weeks of sham or 150 kHz TTFields application.
Average ± standard deviations at study end are shown for control animals versus animals treated with TTFields . (WBC, white blood cells; RBC, red blood cells; HGB, haemoglobin; MCV, mean corpuscular volume; MCH, mean corpuscular haemoglobin; MCHC, MCH concentration.)

## Slide 2
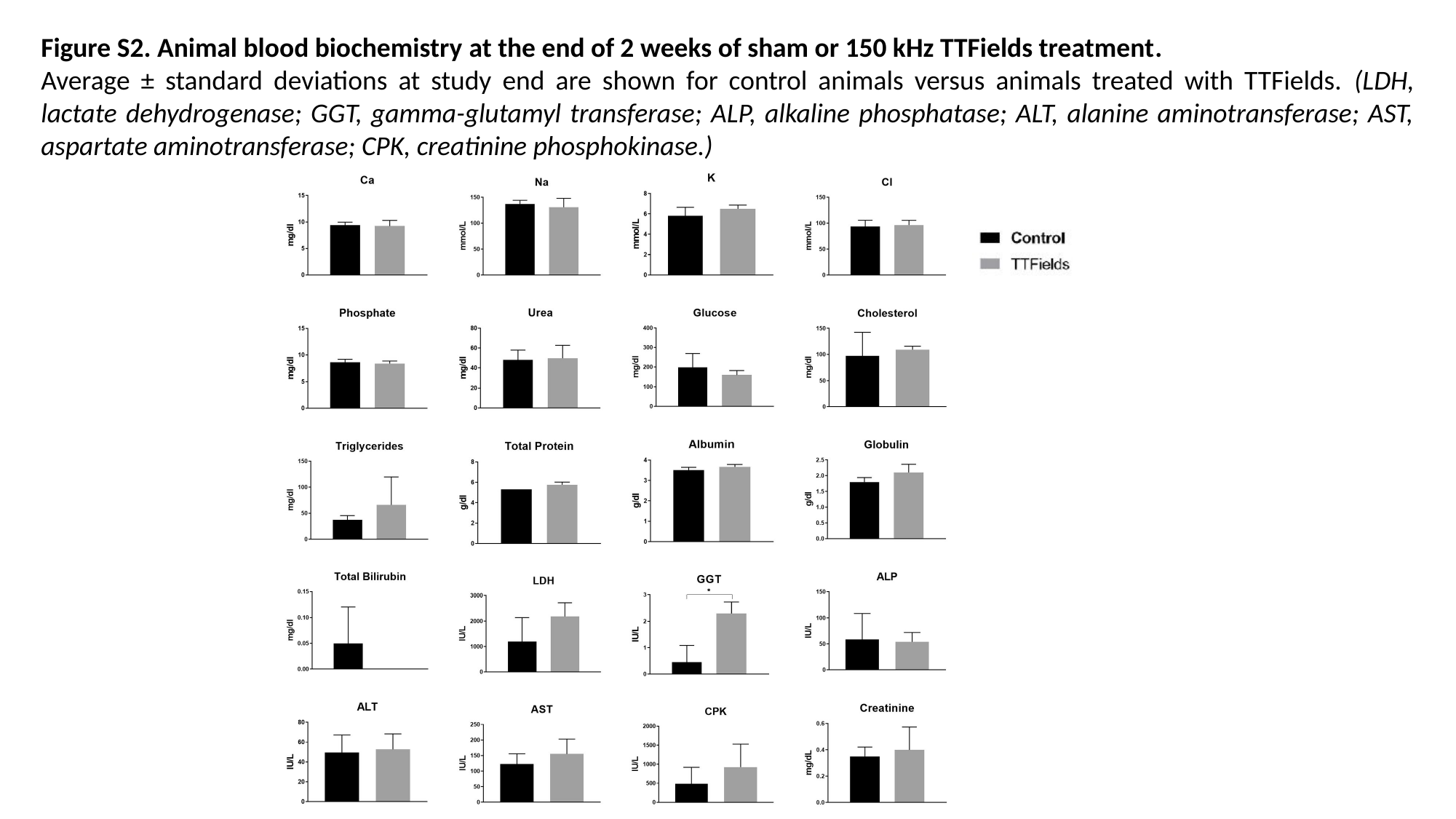

Figure S2. Animal blood biochemistry at the end of 2 weeks of sham or 150 kHz TTFields treatment.
Average ± standard deviations at study end are shown for control animals versus animals treated with TTFields. (LDH, lactate dehydrogenase; GGT, gamma-glutamyl transferase; ALP, alkaline phosphatase; ALT, alanine aminotransferase; AST, aspartate aminotransferase; CPK, creatinine phosphokinase.)

## Slide 3
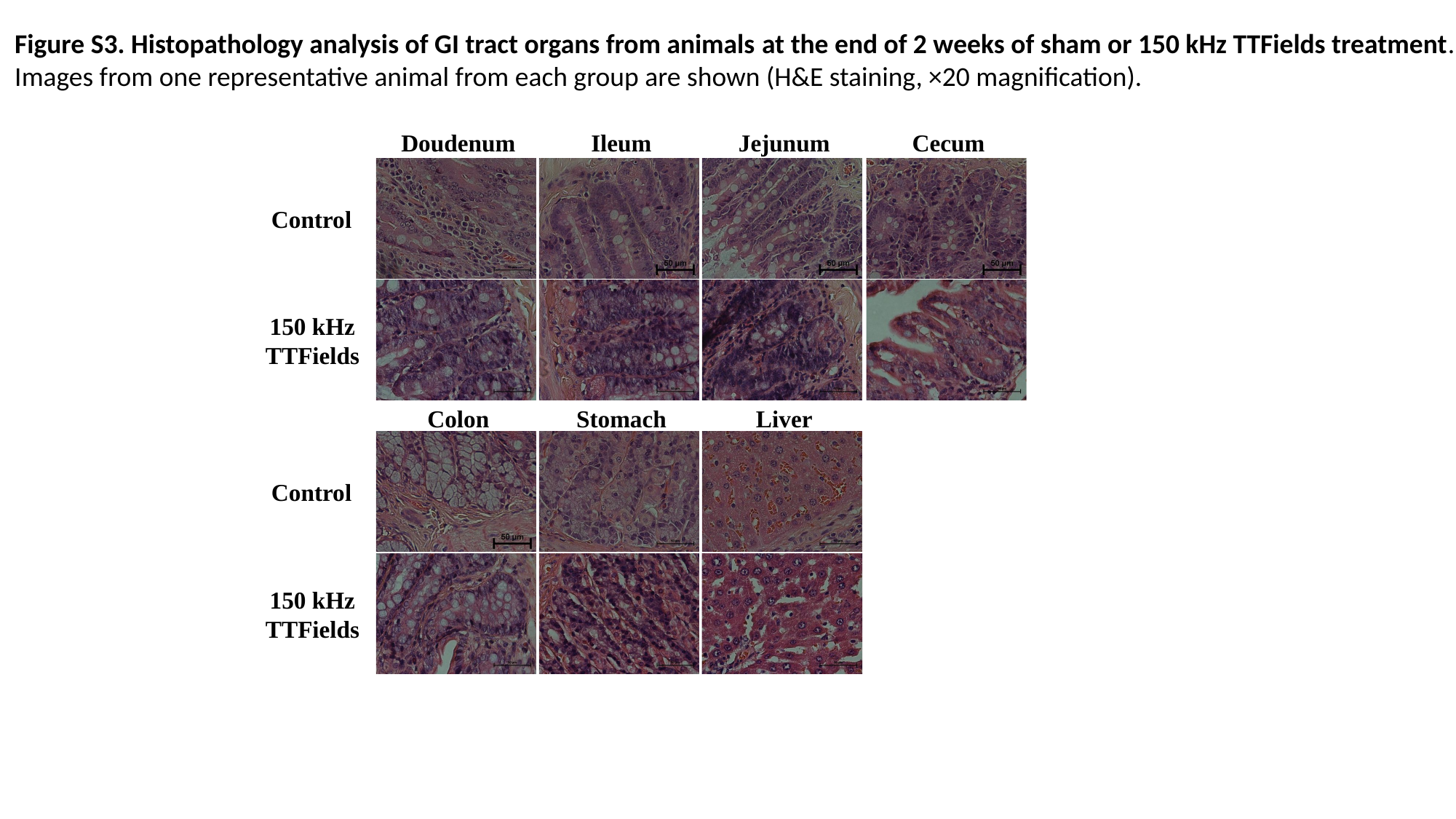

Figure S3. Histopathology analysis of GI tract organs from animals at the end of 2 weeks of sham or 150 kHz TTFields treatment. Images from one representative animal from each group are shown (H&E staining, ×20 magnification).
Doudenum
Ileum
Jejunum
Cecum
Control
150 kHz TTFields
Colon
Stomach
Liver
Control
150 kHz TTFields

## Slide 4
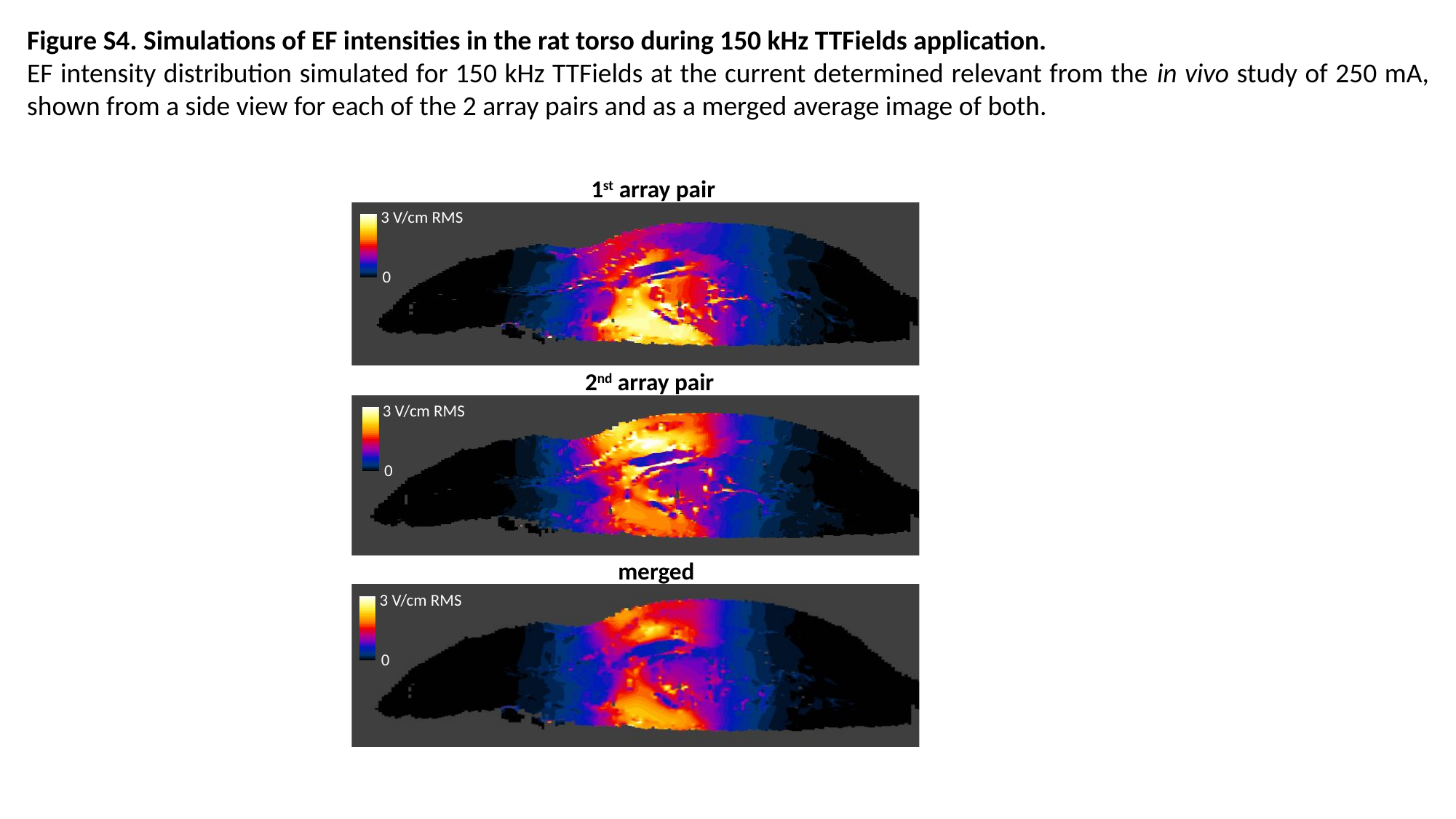

Figure S4. Simulations of EF intensities in the rat torso during 150 kHz TTFields application.
EF intensity distribution simulated for 150 kHz TTFields at the current determined relevant from the in vivo study of 250 mA, shown from a side view for each of the 2 array pairs and as a merged average image of both.
1st array pair
3 V/cm RMS
0
2nd array pair
3 V/cm RMS
0
merged
3 V/cm RMS
0
